# Knowledge and Perception of Pharmacy Interns in Ghana on Antimicrobial Use, Resistance, and Stewardship: A Cross‐Sectional Study

**DOI:** 10.1002/hsr2.72508

**Published:** 2026-05-13

**Authors:** Thelma Ohene‐Agyei, Emmanuella Diane Mankartah, Patrick Adu‐Gyamfi, Ayah Fattal

**Affiliations:** ^1^ Department of Pharmacy Practice and Clinical Pharmacy, School of Pharmacy University of Ghana Legon Accra Ghana

## Abstract

**Background and Aims:**

Antimicrobial resistance (AMR) is a global health crisis, particularly predominant in low‐ and middle‐income countries. Pharmacists play a crucial role in combating AMR, especially through antimicrobial stewardship (AMS) initiatives. In Ghana, the training of pharmacists includes a mandatory 1‐year internship before licensure, aimed at ensuring that graduates are well‐prepared to deliver high‐quality pharmaceutical care. However, the extent to which this training equips interns to address AMR, especially through AMS, remains unclear. This study aimed to determine the knowledge and perceptions of pharmacy interns in Ghana on antimicrobial use (AMU), AMR, and AMS to identify gaps or deficiencies in their training.

**Methods:**

A cross‐sectional study was conducted among 195 pharmacy interns who had just sat for the Ghana Pharmacy Professional Qualifying Examination (GPPQE). A questionnaire adapted from previously published studies was used to collect data. Data were analyzed descriptively using Google Sheets. Knowledge scores were categorized using Bloom's cut‐off criteria: a score of ≥ 80% was considered “good,” while < 80% was classified as “moderate to poor.”

**Results:**

Most interns had received training on AMU (92.3%), AMR (90.3%), and AMS (81.5%). Good knowledge levels were observed for AMU (65.6%) and AMR (58.9%); however, only 43.1% demonstrated good AMS knowledge. Despite 92.3% of interns perceiving the quantity and quality of their knowledge to be good, only 58.0% strongly agreed they had received sufficient training, and 64.6% expressed a desire for further education on AMU, AMR, and AMS, reflecting awareness of training gaps.

**Conclusion:**

Although most interns received training, there were critical knowledge gaps present, particularly in AMS. While interns held positive perceptions of their stewardship roles, a gap exists between their self‐assessed knowledge and objective knowledge performance, especially regarding AMS. Reviewing internship training objectives and harmonizing AMS education across training institutions is recommended to ensure that pharmacy graduates are well prepared for their stewardship roles in practice.

## 1 Introduction

Globally, antimicrobial resistance (AMR) is recognized as a major threat to public health with an estimated 4·71 million deaths associated with AMR and 1.14 million deaths directly attributable to bacterial AMR in 2021 [1]. If no interventions are implemented, it is estimated that by 2050, mortalities attributed to AMR will have increased to 10 million annually [2].

Although AMR is widespread, a disproportionate burden is faced by low and middle‐income countries, with Africa and South Asia bearing the highest burden of deaths [3, 4]. In 2019, sub‐Saharan Africa (SSA) recorded the highest mortality rate from AMR, with 23.5 deaths per 100,000 people [4]. This is linked to factors such as weak regulation of antimicrobial use and constrained healthcare systems [4].

In Ghana, antimicrobial resistance threatens the efficacy of commonly used antimicrobials and endangers the health of the population [5, 6, 7]. Inappropriate use of antimicrobials, driven by factors such as self‐medication, overprescription, and limited public awareness, has accelerated the development of drug‐resistant pathogens [8, 9, 10].

Pharmacists, as key stakeholders in the healthcare system, are uniquely positioned to address the challenges posed by AMR [11, 12]. They promote the optimal use of antimicrobials, monitor and audit prescriptions, ensure medicine quality, and educate health professionals and the public [13]. Their involvement in AMS initiatives reduces inappropriate antimicrobial use, improves clinical outcomes, decreases AMR emergence, and reduces healthcare costs [13, 14].

The training of pharmacists in Ghana is through a 6‐year Doctor of Pharmacy program followed by a mandatory 1‐year internship period before licensure. This internship provides advanced clinical practice experience to ensure that pharmacy graduates are well‐equipped to deliver high‐quality pharmaceutical care. Pharmacy interns, as future pharmacists, represent an important group whose understanding of AMR and AMS will directly influence their ability to contribute to efforts against AMR. However, the extent to which this training prepares interns to address AMR, particularly through AMS initiatives, has not been established.

This needs to be addressed as pharmacist‐led interventions in Ghana have demonstrated promising results in improving antimicrobial stewardship. Studies in hospitals across Sub‐Saharan Africa have shown that pharmacist‐led antimicrobial interventions improve adherence to guidelines and reduce inappropriate prescribing [15]. For example, in Ghana, the use of a mobile app led by pharmacists significantly increased compliance with national prescribing guidelines from 18% to 70% among patients with pneumonia at an outpatient clinic [16]. Despite these successes, existing studies mainly focus on practicing pharmacists and specific settings, leaving a limited understanding of pharmacy interns' knowledge and perceptions regarding antimicrobial resistance and stewardship.

This study, therefore, aims to determine the knowledge and perceptions of pharmacy interns in Ghana on antimicrobial use, resistance, and stewardship.

### 1.1 Aim of Study

The aim of this study is to assess the knowledge and perceptions of pharmacy interns in Ghana on antimicrobial use, resistance, and stewardship.

## 2 Methods

### 2.1 Study Design

A cross‐sectional study was conducted with pharmacy interns who had completed their internship and met the requirements to sit for the Ghana Pharmacy Professional Qualifying Examination (GPPQE).

### 2.2 Study Setting

The survey was administered at the December 2023 GPPQE examination center, held at Zenith University College, Accra, Ghana.

### 2.3 Study Population

The target population comprised all pharmacy interns in Ghana who had completed the 1‐year mandatory internship program and were registered to sit for the December 2023 GPPQE.

### 2.4 Sample Size

The total population comprised 426 interns, as obtained from the official candidate list for the December 2023 GPPQE provided at the examination center. The sample size was subsequently calculated using Yamane's formula: *n* = *N*/(1 + *N* × *e* ^2^) [17], where *n* is the required sample size; *N* is the population size; and *e* is the margin of error (0.05).

A 95% confidence level and a 5% margin of error were assumed. Given a population size of *N* = 426 pharmacy interns and *e* = 0.05, the calculated minimum sample size was 206 participants. A total of 195 fully completed responses were obtained, representing a response rate of approximately 94.7%.

### 2.5 Data Collection and Sampling Technique

Participants were recruited at the examination center using a convenience sampling approach. Data collection was carried out over a 1‐week period at the examination center, with questionnaires administered in person to all consenting candidates following each examination session, covering both the written and oral components. No compensation or incentive was provided to participants for taking part in the study. Participation was voluntary, and all responses were anonymous. Participants were informed of the study's objectives, and written consent was obtained before participation.

### 2.6 Data Collection Instrument

The questionnaire was adapted and modified from similar studies [18, 19, 20] and was pilot‐tested for clarity and relevance of the questions among 10 pharmacy interns. The questionnaire focused on three key areas: (a) knowledge on antimicrobial use (AMU) and antimicrobial resistance (AMR) [19 questions]; (b) knowledge and experience on antimicrobial stewardship (AMS) [19 questions]; and (c) education on antimicrobial use, resistance, and stewardship [14 questions]. Responses were measured using a 5‐point Likert scale; the options were: SA (Strongly Agree), A (Agree), N (Neutral), D (Disagree), and SD (Strongly Disagree). The dependent variables were knowledge, education, and practices regarding antimicrobial use, resistance, and stewardship, while the independent variables included socio‐demographic factors such as age and gender.

### 2.7 Data Analysis

Data collected were input individually and analyzed using Google Sheets (Google LLC, Mountain View, CA, USA). Functions such as COUNTIF, AVERAGE, and STANDARD DEVIATION were used to calculate and produce values. Descriptive statistics were used to summarize the data obtained using frequencies and percentages. The overall score for the knowledge questions was categorized into “good” or “moderate to poor” based on respondents' scores in each domain. A total score of at least ≥ 80% of the maximum obtainable score was considered “good” knowledge, while a score of < 80% was considered “moderate to poor” knowledge. The binary categorization of knowledge scores was adapted from Bloom's cut‐off criteria and other similar studies [18, 20]. The questions that required negative responses were reverse‐scored.

### 2.8 Ethical Approval and Informed Consent Statement

Ethical approval for the study was obtained from the University of Ghana Medical Centre Institutional Review Board (UGMC‐IRB) under protocol ID: UGMC‐IRB/MSRC/004/2023. The questionnaires were distributed after the participants had completed their board examinations, ensuring that participation had no influence on their ability to sit for the examination. Participants were explicitly informed that participation was entirely voluntary and unrelated to the GPPQE. The research team had no affiliation with the examination body and no authority over examination access. All respondents reviewed and signed a written informed consent form prior to participating in the study.

## 3 Results

A total of 195 interns participated in the survey, resulting in a response rate of 94.7%. The mean age of participants was 24.46 years (SD = 2.21). Males slightly outnumbered females, making up 52.3% of the sample.

### 3.1 Training Levels and Sources of Knowledge on Antimicrobial Use, Resistance, and Stewardship

The majority of interns reported having received training, with 92.3% reporting prior training on antimicrobial use and 90.3% on antimicrobial resistance, primarily through lectures (77.9% and 66.2%, respectively) and internships (51.8% and 43.1%) (Table 1). Self‐taught learning played a supplementary role, as indicated by 24.1% and 21.5% of participants. Moreover, 81.5% had also received antimicrobial stewardship training, which was again led by lectures (47.2%) and internships (39.5%).

**Table 1:** Levels and sources of training on antimicrobial use, resistance, and stewardship.

Variable	*n*	%
Training on antimicrobial use?		
Yes	180	92.3
No	15	7.7
If yes, what source?		
Lectures	152	77.9
Internship	101	51.8
Self‐taught	47	24.1
Training on antimicrobial resistance?		
Yes	176	90.3
No	19	9.7
If yes, what source?		
Lectures	129	66.2
Internship	84	43.1
Self‐taught	42	21.5
Training on antimicrobial stewardship		
Yes	159	81.5
No	36	18.5
If yes, what source?		
Lectures	92	47.2
Internship	77	39.5
Self‐taught	36	18.5

### 3.2 Knowledge of Antimicrobial Use

A significant proportion of participants demonstrated awareness of key issues related to antimicrobial misuse and resistance. Moreover, 84.1% agreed or strongly agreed that poor patient counseling can lead to antimicrobial misuse, while 78.5% recognized that prescribers' lack of skills and knowledge contributes to irrational prescribing practices. In addition, 70.8% of participants correctly identified that antimicrobials should only be used when prescribed. However, notable gaps in knowledge were also observed with 42% incorrectly indicating that antibiotics are effective against infections caused by fungi and viruses (Table 2).

**Table 2:** Knowledge of antimicrobial use.

Questions	*N* (%)				
	SA	A	N	D	SD
1. Antibiotics are safe drugs, hence can be commonly used as medication	49 (25.1)	49 (25.1)	28 (14.4)	28 (14.4)	41 (21.0)
2. Antibiotics treat infections from fungi, virus and bacteria	56 (28.7)	26 (13.3)	10 (5.1)	50 (25.7)	53 (27.2)
3. Antimicrobials should only be used when prescribed	138 (70.8)	36 (18.5)	10 (5.1)	4 (2.0)	7 (3.6)
4. Broad‐spectrum antimicrobials are more preferred for the treatment of infections	47 (24.1)	60 (30.8)	46 (23.6)	18 (9.2)	24 (12.3)
5. The body can usually fight mild infections on its own without antimicrobials	85 (43.6)	72 (36.9)	21 (10.8)	7 (3.6)	10 (5.1)
6. Treatment with antimicrobials should be stopped once you feel better	23 (11.8)	12 (6.2)	10 (5.1)	39 (20.0)	111 (56.9)
7. Frequent use of antimicrobials may decrease the efficacy of treatment	106 (54.4)	56 (28.7)	16 (8.2)	7 (3.6)	10 (5.1)
8. Poor counseling of patients can lead to antimicrobial misuse	164 (84.1)	26 (13.4)	3 (1.5)	—	2 (1.0)
9. Poor skills and knowledge of prescribers can cause irrational antimicrobial prescribing	153 (78.5)	36 (18.5)	5 (2.5)	—	1(0.5)
10. Patient self‐medication can increase antimicrobial resistance	164 (84.1)	21 (10.8)	8 (4.1)	—	2 (1.0)
11. Susceptibility tests help to determine the likelihood that a particular antimicrobial will be effective in treatment of a certain microbial infection	158 (81.0)	26 (13.3)	7 (3.6)	3 (1.6)	1 (0.5)

Abbreviations: A, agree; D, disagree; N, neutral; SA, strongly agree; SD, strongly disagree.

### 3.3 Knowledge Score of Antimicrobial Use (AMU)

The average knowledge score was 44.75 ± 4.16, with scores ranging from 0 to 55. The majority (65.6%) demonstrated good knowledge levels, while 34.4% exhibited moderate to poor knowledge levels.

### 3.4 Knowledge of Antimicrobial Resistance (AMR)

Participants demonstrated a good understanding of AMR (Table 3). Encouragingly, 83.1% of participants acknowledged their role as pharmacy interns in decreasing the prevalence of AMR. However, some misconceptions were evident, as 60% incorrectly believed that “people can become resistant to antimicrobials,” rather than recognizing that resistance develops in microorganisms.

**Table 3:** Knowledge of antimicrobial resistance (AMR).

Questions	*N* (%)				
	SA	A	N	D	SD
1. Microorganisms can become resistant to antimicrobials	166 (85.1)	21 (10.8)	4 (2.1)	1 (0.5)	3 (1.5)
2. People can become resistant to antimicrobials	81 (41.5)	36 (18.5)	21 (10.8)	24 (12.3)	33 (16.9)
3. The more antimicrobials are used, the higher the risk of resistance that may develop	137 (70.3)	31 (15.9)	18 (9.2)	2 (1.0)	7 (3.6)
4. Non‐compliance does not contribute to the development of antimicrobial resistance	32 (16.4)	13 (6.7)	7 (3.6)	40 (20.5)	103 (52.8)
5. Resistance can spread from animals to humans	119 (61.0)	38 (19.5)	23 (11.8)	6 (3.1)	9 (4.6)
6. Resistance can spread from person to person	84 (43.1)	42 (21.5)	35 (17.9)	12 (6.2)	22 (11.3)
7. Taking antimicrobials correctly may reduce the risk of antimicrobial resistance	142 (72.8)	38 (19.5)	8 (4.1)	2 (1.0)	5 (2.6)
8. Antimicrobials should be stopped when the patient has clinically improved to reduce the risk of resistance	54 (27.7)	19 (9.7)	21 (10.8)	31 (15.9)	70 (35.9)
9. As a pharmacy intern and also a member of the society, I have a role to play in decreasing the prevalence of antimicrobial resistance	162 (83.1)	30 (15.4)	2 (1.0)	—	1 (0.5)

Abbreviations: A, agree; D, disagree; N, neutral; SA, strongly agree; SD, strongly disagree.

The average knowledge score on AMR was 35.82 ± 3.81 (range: 0–45). 58.9% of participants demonstrated good knowledge levels, and 41.1% demonstrated moderate to poor knowledge levels.

### 3.5 Knowledge of Antimicrobial Stewardship (AMS)

70.8% of study participants strongly agreed that pharmacists “promote optimal use of antimicrobial agents,” and 61.5% strongly agreed that one goal of AMS is to “minimize toxicity and other adverse effects,” highlighting good awareness of patient safety.

However, several misconceptions were present as 34.3% believed AMS is “a study of only antibiotics,” and 30.3% of respondents either strongly agreed or agreed with the statement “The role of AMS is to encourage over‐the‐counter prescription of antimicrobial agents.” Misunderstandings about AMS team composition were also apparent, as 60.5% agreed that “occupational therapists” are part of the AMS team. Gaps in knowledge regarding the goals of AMS were evident as 21.5% strongly agreed that AMS seeks to “increase antimicrobial use” and 20.5% strongly agreed that AMS aims to “increase the use of broad‐spectrum antimicrobials” (Table 4).

**Table 4:** Knowledge of antimicrobial stewardship (AMS).

Questions	*N* (%)				
	SA	A	N	D	SD
1. AMS is a program that increases the treatment duration to ensure therapeutic efficacy	64 (32.8)	40 (20.5)	32 (16.4)	19 (9.8)	40 (20.5)
2. AMS is a study of only antibiotics	67 (34.3)	59 (30.3)	32 (16.4)	17 (8.7)	20 (10.3)
3. AMS is a process that involves suitable antimicrobial dosing and route of administration	101 (51.8)	67 (34.4)	20 (10.3)	4 (2.0)	3 (1.5)
4. AMS is a process that involves monitoring the duration of antimicrobial therapy	101 (51.8)	67 (34.4)	17 (8.7)	3 (1.5)	7 (3.6)
5. The role of AMS is to encourage over‐the‐counter prescription of antimicrobial agents	36 (18.5)	23 (11.8)	17 (8.7)	29 (14.9)	90 (46.1)
*Goals of antimicrobial stewardship seek to achieve*					
6. Increasing antimicrobial use	42 (21.5)	45 (23.1)	26 (13.3)	31 (15.9)	51 (26.2)
7. Reducing hospital stay	87 (44.6)	53 (27.2)	24 (12.3)	16 (8.2)	15 (7.7)
8. Increasing duration of therapy to ensure therapeutic efficacy	38 (19.5)	34 (17.4)	46 (23.6)	27 (13.9)	50 (25.6)
9. Increasing use of broad‐spectrum antimicrobials	40 (20.5)	31 (15.9)	37 (19.0)	30 (15.4)	57 (29.2)
10. Minimizing toxicity and other adverse effects	120 (61.5)	54 (27.7)	12 (6.2)	7 (3.6)	2 (1.0)
*Members of the antimicrobial stewardship team include*					
11. Infectious disease physicians	130 (66.7)	46 (23.6)	15 (7.7)	1 (0.5)	3 (1.5)
12. Occupational therapists	84 (43.1)	34 (17.4)	56 (28.7)	9 (4.6)	12 (6.2)
13. Clinical and hospital pharmacists	147 (75.4)	36 (18.5)	10 (5.1)	1 (0.5)	1 (0.5)
14. Infection control staff	137 (70.3)	39 (20.0)	15 (7.7)	—	4 (2.0)
15. Hospital cleaning staff	91 (46.7)	40 (20.5)	33 (16.9)	14 (7.2)	17 (8.7)
*The role of the pharmacist in antimicrobial stewardship*					
16. Promote optimal use of antimicrobial agents	138 (70.8)	38 (19.5)	9 (4.6)	1 (0.5)	9 (4.6)
17. Prescribe antimicrobial agents over‐the‐counter	46 (23.6)	24 (12.3)	17 (8.7)	27 (13.9)	81 (41.5)
18. Educate healthcare professionals	150 (76.9)	34 (17.5)	6 (3.1)	3 (1.5)	2 (1.0)
19. Work with therapeutic committees to develop policies	142 (72.8)	42 (21.6)	8 (4.1)	2 (1.0)	1 (0.5)

Abbreviations: A, agree; D, disagree; N, neutral; SA, strongly agree; SD, strongly disagree.

The majority (56.9%) of participants demonstrated moderate to poor knowledge scores, while 43.1% exhibited good knowledge levels. The overall average knowledge score was 70.04 ± 8.99, with scores ranging from 0 to 95.

### 3.6 Perception of Interns on Their Knowledge on Antimicrobial Use, Resistance, and Stewardship

The majority of respondents demonstrated confidence in their knowledge on AMR, with 73.3% strongly agreeing that they understand what it is. Additionally, 65.6% strongly agreed they have the necessary information to educate others about the prudent use of antimicrobials and antimicrobial resistance. Despite these strengths, participants expressed uncertainty about the adequacy of their training. Only 58.0% strongly agreed that they had received sufficient training on antimicrobial use, resistance, and stewardship, and only 58.5% strongly agreed that they had sufficient knowledge about how to use antimicrobials appropriately in their future practice.

Participants also demonstrated awareness of the importance of continuing education; a notable proportion (64.6%) strongly agreed that “it is necessary for me to get more information about antimicrobials.” Similar percentages were observed for desires for further education on appropriate AMU (64.6%), AMR (63.1%), and AMS (66.2%).

Confidence levels varied, with 53.8% strongly agreeing that they feel confident about their knowledge and future practice in antimicrobial prescribing, while 39.5% agreed, and 6.2% remained neutral. Participants also recognized the broader benefits of AMS, with 70.3% strongly agreeing that it improves the cost‐effectiveness of healthcare sectors (Table 5).

**Table 5:** Perception of interns on the quantity and quality of their knowledge on antimicrobial use, resistance, and stewardship.

Questions	*N* (%)				
	SA	A	N	D	SD
1. I know what antimicrobial resistance is	143 (73.3)	50 (25.7)	2 (1.0)	—	—
2. I know what information to give to individuals about the prudent use of antimicrobials and antimicrobial resistance	128 (65.6)	63 (32.3)	4 (2.1)	—	—
3. I believe I have adequate training on antimicrobial use, resistance, and stewardship from my training in the university	113 (58.0)	64 (32.8)	16 (8.2)	—	2 (1.0)
4. I have sufficient knowledge about how to use antimicrobials appropriately for my future practice	114 (58.5)	71 (36.4)	9 (4.6)	1 (0.5)	—
5. I think it is necessary for me to get more information about antimicrobials	126 (64.6)	57 (29.2)	11 (5.7)	1 (0.5)	—
6. I feel confident about my knowledge and future practice in the area of antimicrobial prescribing	105 (53.8)	77 (39.5)	12 (6.2)	1 (0.5)	—
7. I think antimicrobial stewardship will improve the patient's clinical outcomes	142 (72.8)	47 (24.1)	4 (2.1)	1 (0.5)	1 (0.5)
8. I think antimicrobial stewardship will reduce antimicrobial resistance	144 (73.8)	40 (20.5)	6 (3.1)	2 (1.0)	3 (1.5)
9. I think antimicrobial stewardship will improve the cost‐effectiveness of healthcare sectors	137 (70.3)	44 (22.5)	9 (4.6)	1 (0.5)	4 (2.1)
10. I think antimicrobial stewardship will improve the collaboration between healthcare providers	123 (63.1)	62 (31.8)	7 (3.6)	1 (0.5)	2 (1.0)
11. A strong knowledge of antimicrobial resistance and stewardship is important for future career	139 (71.3)	43 (22.1)	11 (5.6)	1 (0.5)	1 (0.5)
12. I would like more education on appropriate use of antimicrobials	126 (64.6)	58 (29.8)	10 (5.1)	1 (0.5)	—
13. I would like more education on antimicrobial resistance	123 (63.1)	59 (30.3)	11 (5.6)	2 (1.0)	—
14. I would like more education on antimicrobial stewardship	129 (66.2)	55 (28.2)	8 (4.1)	1 (0.5)	2 (1.0)

Abbreviations: A, agree; D, disagree; N, neutral; SA, strongly agree; SD, strongly disagree.

The results indicate that interns generally perceive their knowledge of antimicrobial use, resistance, and stewardship to be good. The average score was 64.21 ± 6.42, with scores ranging from 0 to 75. A significant majority (92.3%) exhibited good perception levels regarding the quantity and quality of their knowledge, while only a small percentage (7.7%) fell into the low to moderate perception category.

## 4 Discussion

The key findings of this study indicate that while the majority of interns demonstrated good theoretical knowledge of AMU (65.6%) and AMR (58.9%), their knowledge level of AMS (43.1%) and their confidence in applying stewardship principles in practice were comparatively weaker. This suggests that although the pharmacy education system in Ghana is effective in equipping students with solid knowledge of AMU and AMR, greater emphasis is necessary to strengthen the knowledge and practical application of AMS principles.

The high knowledge scores in AMU and AMR knowledge likely reflect the training exposure provided during undergraduate years through lectures and internships, as reported by 92.3% and 90.3% of participants for AMU and AMR, respectively. Similar results have also been reported in other low‐ and middle‐income countries (LMICs), where structured academic teaching, internships, and public awareness campaigns have improved understanding of AMR among health professional students, which reflects a shared public health challenge in AMR and a common emphasis on AMR during training in LMICs [18, 21, 22, 23].

Despite the high knowledge scores, notable misconceptions persisted. Over half of the participants (53.8%) incorrectly believed that antibiotics are effective against fungal and viral infections, and nearly half (49.2%) considered antibiotics safe for common use. These misconceptions are consistent with findings by [24], which found that over 57% of junior pharmacy students in Sri Lanka holding the misconception that antibiotics are effective against viral infections. The study by [25] also corroborates our findings, reporting that 22% of pharmacy students held the misconception that antibiotics are safe for common use. In addition, two in five interns (41.5%) incorrectly believed that people, rather than bacteria, become resistant to antimicrobials.

This misunderstanding has been documented in similar studies and is often attributed to oversimplified messaging in educational materials or public health campaigns [26, 27]. Furthermore, 27.7% agreed that antimicrobials should be stopped when patients improve, contradicting guidelines advocating for completing prescribed courses to prevent resistance. This misconception likely stems from insufficient emphasis on adherence during training, highlighting the need for targeted education on adherence [28].

Fewer interns reported high AMS knowledge scores compared to AMU and AMR. These disparities in technical knowledge on AMS, despite overall high awareness of AMU and AMR, may be attributed to the variations in information sources, the lack of a structured AMS curriculum‐based training, and gaps in practical training.

The WHO has highlighted that although most countries now have national action plans to address AMR, the quality and coverage of education and training initiatives remain uneven. This underscores the need for standardized educational resources that draw on global evidence and best practices while remaining adaptable to local contexts [29].

Despite Ghana's National Action Plan on Antimicrobial Resistance prioritizing education and training, significant gaps remain in structured AMS education. The plan explicitly calls for the training and re‐training of core clinical care providers on the responsible use of antimicrobials across all sectors and mandates that training institutions incorporate antimicrobial stewardship into pre‐service curricula for health and related professionals. It further set an indicator to increase the proportion of health training institutions that include AMR modules in their curricula [30]. However, evidence shows that integration of AMS into pre‐service training in Ghana has not been systematic. Regional capacity‐building analyses highlight reliance on workshops and ad hoc CPD activities, with limited incorporation of AMS into formal training curricula of health professionals [31]. This gap between policy objectives and training implementation shows the need for stronger enforcement of curriculum reforms and sustainable, multidisciplinary AMS education frameworks.

Given that pharmacy schools in Ghana do not have a unified curriculum, there is the need for a comparative evaluation of AMS‐related training across institutions. International frameworks already provide a foundation for addressing this gap. The WHO Competency Framework for Health Workers' Education and Training on AMR, for instance, offers a structured approach for both pre‐service and in‐service training, promoting consistent stewardship competencies worldwide [29]. The UK further demonstrates the feasibility of this approach, where a national consensus on undergraduate AMS competencies has established a common foundation for curriculum design across professions [32]. Evidence from interprofessional education further supports this harmonization, showing that structured AMS curricula that combine independent learning with interprofessional workshops can significantly enhance learners' knowledge and attitudes toward stewardship [33]. The delivery of AMS education must also adopt a comprehensive, structured, multidisciplinary, and hands‐on approach to improve knowledge transfer and its practical application in clinical settings [23, 34, 35].

Drawing on these frameworks and evidence, the Pharmacy Council of Ghana, as the statutory body responsible for regulating pharmacy education and practice under the Health Professions Regulatory Bodies Act, 2013 (Act 857), is mandated to ensure that pharmacy education and training meet required standards and to accredit pharmacy programs (Sections 80(g) and 80(i)) [36]. These functions position the Council to lead the harmonization of key competencies, including antimicrobial stewardship, across pharmacy training institutions. Through this authority, the Council can direct all training institutions to adopt a unified, competency‐based AMS curriculum as part of the pre‐service undergraduate program, ensuring that all pharmacy graduates enter their internship with a consistent and adequate foundation in AMS.

Another key finding of this study was the high overall perception scores among interns, with 92.3% exhibiting good perception levels regarding the quantity and quality of their knowledge on AMU, AMR, and AMS. The majority of interns expressed confidence in their understanding of antimicrobial resistance, their ability to educate others, and their readiness for future practice. However, these self‐reported perceptions contrasted with the objective knowledge scores, particularly the comparatively lower AMS knowledge score of 43.1%. This points to a gap between perceived and actual competence.

Such gaps between self‐assessment and objective performance have been documented elsewhere.

Final‐year pharmacy students in Saudi Arabia similarly reported relatively high confidence in their AMS and antibiotic‐related competence despite only about half achieving good knowledge scores [21]. In East Africa, only 36.6% of final‐year medical and pharmacy students demonstrated good overall knowledge, and large proportions could not select appropriate antibiotics, doses, or determine when to switch from intravenous to oral therapy [23]. Similar confidence–competence gaps have been reported among pharmacy students in Malaysia and Nigeria. While Malaysian students had greater exposure to AMS and demonstrated higher knowledge of appropriate antibiotic therapy, Nigerian students reported higher levels of self‐confidence, suggesting that confidence does not necessarily reflect actual competence [37]. This pattern was similarly observed among Sudanese pharmacy students, one‐third of whom lacked confidence in interpreting microbiology results despite demonstrating substantial AMS and AMR knowledge [38].

Nonetheless, there were encouraging signs; the majority of interns in this study recognized the value of AMS, with over 70% strongly agreeing that it would improve patient clinical outcomes, reduce AMR, and enhance cost‐effectiveness in healthcare, consistent with positive AMS attitudes reported across multiple low‐ and middle‐income settings [21, 38, 39, 40]. Furthermore, over 63% of interns expressed a desire for more training across all three domains, reflecting awareness of their own training gaps and a receptiveness to further learning. Comparable proportions of pharmacy students in Northern Nigeria, Malaysia, and India have similarly requested further teaching on antibiotics, AMR, and AMS, with fewer than half feeling adequately prepared for future practice [37, 39, 41]. These perceptions align with findings from other LMICs, where pharmacy students and interns tend to recognize their professional role in tackling AMR while simultaneously identifying inadequacies in their preparation [39, 40, 41].

## 5 Conclusion

Taken together, these findings highlight the need for targeted educational reforms to strengthen antimicrobial stewardship competencies among pharmacy trainees in Ghana. The findings indicate that although most interns acknowledge their role in combating antimicrobial resistance as future healthcare professionals and possess a strong theoretical understanding of antimicrobial use (AMU) and resistance (AMR), their understanding of antimicrobial stewardship (AMS) principles requires further development, particularly regarding the broader goals of AMS and appropriate antimicrobial prescribing practices. While interns held positive perceptions of AMS and expressed confidence in their stewardship roles, a notable gap exists between this self‐assessed confidence and their objective knowledge performance. Nevertheless, their positive disposition toward AMS and desire for further training provide a strong foundation for future educational interventions. Reviewing internship objectives across various universities to assess their impact on AMS training and a consequent improvement and harmonization of AMS education across all the training institutions will ensure that all pharmacy graduates are fully prepared for their stewardship roles in clinical practice.

## 6 Recommendations

Based on the gaps identified in this study, several targeted actions are recommended. Firstly, the Pharmacy Council of Ghana should prioritize the development and enforcement of a harmonized, competency‐based AMU, AMR, and AMS curricula across all pharmacy training institutions, drawing on established international frameworks such as the WHO Competency Framework for Health Workers' Education and Training on AMR. To address the low confidence in applying AMS principles in clinical settings, training curricula should incorporate more hands‐on, case‐based learning with clinical rotations and seminars focusing on AMS. Additionally, more educational resources, such as comprehensive guides, online courses, webinars, and interactive sessions, should be provided to keep students up‐to‐date with international AMS guidelines.

## 7 Limitations

The cross‐sectional nature of the study implies that causality cannot be inferred, and the study may be susceptible to recall bias. The study utilized surveys as a data collection method, which may not fully capture the complexities of interns' perceptions and experiences. The use of convenience sampling may have introduced selection bias, as only interns who were interested in the study were included. This may limit the representativeness of the sample; hence, the findings should be interpreted with caution when generalizing beyond the study cohort. Additionally, the reliance on self‐reported data is a potential source of social desirability bias, whereby participants may have over‐ or under‐reported their knowledge and perceptions. To mitigate this, participation was entirely voluntary, and all responses were anonymous, which was communicated to participants prior to data collection. A qualitative study exploring the enablers and barriers to antimicrobial use and stewardship among pharmacy interns will provide further relevant information.

## 8 Generalizability

The GPPQE is a national examination sat by all eligible pharmacy interns in Ghana at a single venue. The study recruited directly from this sitting and achieved a response rate of 94.7%. The findings can therefore be considered representative of the 2023 national cohort of pharmacy interns in Ghana. However, as the study captures a single point in time, caution is warranted in extrapolating these findings beyond this cohort. Variations in training quality or shifts in knowledge and perceptions across preceding or subsequent cohorts cannot be accounted for.

## Author Contributions

**Thelma Ohene‐Agyei:** conceptualization, writing – review and editing, supervision, methodology. **Emmanuella Diane Mankartah:** writing – original draft, writing – review and editing, formal analysis, data curation, validation. **Patrick Adu‐Gyamfi:** conceptualization, writing – review and editing, methodology. **Ayah Fattal:** methodology, data curation, validation, writing – original draft.

## Funding

The authors have nothing to report.

## Conflicts of Interest

The authors declare no conflicts of interest.

## Transparency Statement

The lead author, Thelma Ohene‐Agyei, affirms that this manuscript is an honest, accurate, and transparent account of the study being reported; that no important aspects of the study have been omitted; and that any discrepancies from the study as planned (and, if relevant, registered) have been explained.

## Supporting information

Supporting File 1

Supporting File 2

## Data Availability

The data that support the findings of this study are available in the supporting information of this article.
