# Supplementary material for: Knowledge and Perception of Pharmacy Interns in Ghana on Antimicrobial Use, Resistance, and Stewardship: A Cross‐Sectional Study
Source: Health Sci Rep. 2026 May 13;9(5):e72508. doi: 10.1002/hsr2.72508 (PMC13169130; doi:10.1002/hsr2.72508)
Supplement: Supplementary file 1 — Supporting File 1 [file HSR2-9-e72508-s002.docx]

QUESTIONNAIRE

TOPIC: KNOWLEDGE AND PERSPECTIVES OF PHARMACY INTERNS IN GHANA ON ANTIMICROBIAL USE, RESISTANCE AND STEWARDSHIP

SECTION A: SOCIO-DEMOGRAPHIC INFORMATION

Indicate the appropriate answer

**1. Gender**  Male [ ]  Female [ ]

**2. Age**  18-21 [ ]  22-25 [ ]  >25 [ ]

| Have you obtained any training on: | Yes | No | If yes, where did you obtain this? Tick as appropriate. | | | |
| --- | --- | --- | --- | --- | --- | --- |
|  |  |  | Lectures from university | Internship | Self-taught | Other (Please specify) |
| **Antimicrobial use**? |  |  |  |  |  |  |
| **Antimicrobial resistance**? |  |  |  |  |  |  |
| **Antimicrobial stewardship**? |  |  |  |  |  |  |

SECTION B: ANTIMICOBIAL USE AND RESISTANCE

Knowledge on antimicrobial use (Please tick appropriate response)

Key: SA= Strongly Agree; A= Agree; N= Neutral; SD= Strongly Disagree; D= Disagree

|  | SA | A | N | D | SD |
| --- | --- | --- | --- | --- | --- |
| Antibiotics are safe drugs, hence can be commonly used as medication |  |  |  |  |  |
| Antibiotics treat infections from fungi, virus and bacteria |  |  |  |  |  |
| Antimicrobials should only be used when prescribed |  |  |  |  |  |
| Broad spectrum antimicrobials are more preferred for the treatment of infections |  |  |  |  |  |
| The body can usually fight mild infections on its own without antimicrobials |  |  |  |  |  |
| Treatment with antimicrobials should be stopped once you feel better |  |  |  |  |  |
| Frequent use of antimicrobials may decrease the efficacy of treatment |  |  |  |  |  |
| Poor counselling of patients can lead to antimicrobial misuse |  |  |  |  |  |
| Poor skills and knowledge of prescribers can cause irrational antimicrobial prescribing |  |  |  |  |  |
| Patient self-medication can increase antimicrobial resistance |  |  |  |  |  |
| Susceptibility tests helps to determine the likelihood that a particular antimicrobial will be effective in treatment of a certain microbial infection |  |  |  |  |  |

Knowledge on antimicrobial resistance (Please tick appropriate response)

SA= Strongly Agree; A= Agree; N= Neutral; SD= Strongly Disagree; D= Disagree

|  | SA | A | N | D | SD |
| --- | --- | --- | --- | --- | --- |
| Microorganisms can become resistant to antimicrobials. |  |  |  |  |  |
| People can become resistant to antimicrobials |  |  |  |  |  |
| The more antimicrobials are used, the higher the risk of resistance that may develop |  |  |  |  |  |
| Non-compliance does not contribute to the development of antimicrobial resistance |  |  |  |  |  |
| Resistance can spread from animals to humans |  |  |  |  |  |
| Resistance can spread from person to person |  |  |  |  |  |
| Taking antimicrobials correctly may reduce the risk of antimicrobial resistance |  |  |  |  |  |
| Antimicrobials should be stopped when the patient has clinically improved to reduce the risk of resistance |  |  |  |  |  |
| As a pharmacy intern and also a member of the society, I have a role to play in decreasing the prevalence of antimicrobial resistance |  |  |  |  |  |

SECTION C: KNOWLEDGE AND EXPERIENCE ON ANTIMICROBIAL STEWARDSHIP (AMS)

KEY: SA= Strongly Agree; A= Agree; N= Neutral; SD= Strongly Disagree; D= Disagree

|  | SA | A | N | D | SD |
| --- | --- | --- | --- | --- | --- |
| AMS is a program that increases the treatment duration to ensure therapeutic efficacy |  |  |  |  |  |
| AMS is a study of only antibiotics |  |  |  |  |  |
| AMS is a process that involves a suitable antimicrobial dosing and route of administration |  |  |  |  |  |
| AMS is a process that involves monitoring the duration of antimicrobial therapy |  |  |  |  |  |
| The role of AMS is to encourage over the counter prescription of antimicrobial agents |  |  |  |  |  |
| **Goals of antimicrobial stewardship seek to achieve:** |  | | | | |
| Increasing antimicrobial use |  |  |  |  |  |
| Reducing hospital stay |  |  |  |  |  |
| Increasing duration of therapy to ensure therapeutic efficacy |  |  |  |  |  |
| Increasing use of broad spectrum antimicrobials |  |  |  |  |  |
| Minimizing toxicity and other adverse effects |  |  |  |  |  |
| **Members of the antimicrobial stewardship team include** |  | | | | |
| Infectious disease physicians |  |  |  |  |  |
| Occupational therapists |  |  |  |  |  |
| Clinical and hospital pharmacists |  |  |  |  |  |
| Infection control staff |  |  |  |  |  |
| Hospital cleaning staff |  |  |  |  |  |
| **The role of the pharmacist in antimicrobial stewardship:** |  | | | | |
| Promote optimal use of antimicrobial agents |  |  |  |  |  |
| Prescribe antimicrobial agents over-the-counter |  |  |  |  |  |
| Educate healthcare professionals |  |  |  |  |  |
| Work with therapeutic committees to develop policies |  |  |  |  |  |

SECTION D: EDUCATION ON ANTIMICROBIAL USE, RESISTANCE AND STEWARDSHIP

|  | SA | A | N | D | SD |
| --- | --- | --- | --- | --- | --- |
| I know what antimicrobial resistance is |  |  |  |  |  |
| I know what information to give to individuals about the prudent use of antimicrobials and antimicrobial resistance |  |  |  |  |  |
| I believe I have adequate training on antimicrobial use, resistance and stewardship from my training in the university |  |  |  |  |  |
| I have sufficient knowledge about how to use antimicrobials appropriately for my future practice |  |  |  |  |  |
| I think it is necessary for me to get more information about antimicrobials |  |  |  |  |  |
| I feel confident about my knowledge and future practice in the area of antimicrobial prescribing |  |  |  |  |  |
| I think antimicrobial stewardship will improve the patient’s clinical outcomes |  |  |  |  |  |
| I think antimicrobial stewardship will reduce antimicrobial resistance |  |  |  |  |  |
| I think antimicrobial stewardship will improve the cost-effectiveness of healthcare sectors |  |  |  |  |  |
| I think antimicrobial stewardship will improve the collaboration between healthcare providers |  |  |  |  |  |
| A strong knowledge of antimicrobial resistance and stewardship is important for future career |  |  |  |  |  |
| I would like more education on appropriate use of antimicrobials |  |  |  |  |  |
| I would like more education on antimicrobial resistance |  |  |  |  |  |
| I would like more education on antimicrobial stewardship |  |  |  |  |  |
